# Implementing pelvic floor muscle training for women with pelvic organ prolapse: a realist evaluation of different delivery models

**DOI:** 10.1186/s12913-020-05748-8

**Published:** 2020-10-01

**Authors:** Purva Abhyankar, Joyce Wilkinson, Karen Berry, Sarah Wane, Isabelle Uny, Patricia Aitchison, Edward Duncan, Eileen Calveley, Helen Mason, Karen Guerrero, Douglas Tincello, Doreen McClurg, Andrew Elders, Suzanne Hagen, Margaret Maxwell

**Affiliations:** 1 Faculty of Health Sciences and Sport, University of Stirling, Stirling, FK9 4LA UK; 2 Nursing, Midwifery and Allied Health Professions Research Unit, University of Stirling, Stirling Innovation Park, Stirling, FK9 4NF UK; 3 Department of Sport, Exercise and Rehabilitation, University of Northumbria, Sutherland Building, Newcastle-upon-Tyne, NE1 8ST UK; 4 Yunus Centre for Social Business and Health, Glasgow Caledonian University, Cowcaddens Road, Glasgow, G4 0BA UK; 5 Queen Elizabeth University Hospital, 1345 Govan Road, Glasgow, G51 4TF UK; 6 Department of Health Sciences, University of Leicester, Centre for Medicine, University Road, Leicester, LE1 RRH UK; 7 Nursing, Midwifery and Allied Health Professions Research Unit, Glasgow Caledonian University, Govan Mbeki Building, Cowcaddens Road, Glasgow, G4 0BA UK

**Keywords:** Implementation science, Realist evaluation, Prolapse, Context, Pelvic floor muscle training, Health service delivery, Role expansion, Inter-professional working, Professional identity

## Abstract

**Background:**

Pelvic Floor Muscle Training (PFMT) has been shown to be effective for pelvic organ prolapse in women, but its implementation in routine practice is challenging due to lack of adequate specialist staff. It is important to know if PFMT can be delivered by different staff skill mixes, what barriers and facilitators operate in different contexts, what strategies enable successful implementation and what are the underlying mechanisms of their action. PROPEL intervention was designed to maximise the delivery of effective PFMT in the UK NHS using different staff skill mixes. We conducted a realist evaluation (RE) of this implementation to understand what works, for whom, in what circumstances and why.

**Methods:**

Informed by the Realist and RE-AIM frameworks, the study used a longitudinal, qualitative, multiple case study design. The study took place in five, purposively selected, diverse NHS sites across the UK and proceeded in three phases to identify, test and refine a theory of change. Data collection took place at 4 time points over an 18 month implementation period using focus groups and semi-structured interviews with a range of stakeholders including service leads/managers, senior practitioners, newly trained staff and women receiving care in the new service models. Data were analysed using thematic framework approach adapted to identify Context, Mechanism and Outcome (CMO) configurations of the RE.

**Results:**

A heightened awareness of the service need among staff and management was a mechanism for change, particularly in areas where there was a shortage of skilled staff. In contrast, the most established specialist physiotherapist-delivered PFMT service activated feelings of role protection and compromised quality, which restricted the reach of PFMT through alternative models. Staff with some level of prior knowledge in women’s health and adequate organisational support were more comfortable and confident in new role. Implementation was seamless when PFMT delivery was incorporated in newly trained staff’s role and core work.

**Conclusion:**

Roll-out of PFMT delivery through different staff skill mixes is possible when it is undertaken by clinicians with an interest in women’s health, and carefully implemented ensuring adequate levels of training and ongoing support from specialists, multi-disciplinary teams and management.

## Background

Pelvic organ prolapse (POP) is a common urogenital condition affecting up to 50% of parous women over 40 years of age [1, 2]. Treatments include corrective surgery and conservative treatments such as pelvic floor muscle training (PFMT) and vaginal pessaries [3]. A significant proportion of women undergo surgical interventions, which can be prone to complications and/or failure, often requiring remedial or repeat surgeries [4, 5]. In recent years surgical implants with polypropylene mesh have been frequently used as a treatment for POP. These ‘mesh implants’ are now known to be associated with significant complications such as pain and erosion [3, 5], resulting in their use being suspended in Scotland in 2014 [3] and in the rest of the UK in 2018. However, awareness of POP and conservative treatment options is poor among women [6] and healthcare professionals [7, 8].

PFMT is a conservative treatment that has been shown to be clinically and cost effective for management of POP (POPPY trial) [7] and has been recommended as a first line of treatment in the recently updated NICE guidelines [9]. It involves a structured and individualized program of exercises that aims to improve pelvic floor muscle strength, endurance, power, relaxation, or a combination of these parameters [10]. PFMT is usually delivered by specialist physiotherapists who are trained in appropriate assessment of prolapse and training women to perform pelvic floor muscle exercises tailored to their need, although there is potential for involvement of continence nurses, gynaecologists and primary care practitioners who have pelvic health within their scope of practice.

However, knowledge of efficacy and effectiveness of interventions is rarely sufficient to ensure adoption and implementation into routine clinical practice. An important challenge to implementing evidence-based PFMT into practice is the limited and varied availability of specialist women’s health physiotherapists to deliver the intervention to large numbers of women in the UK. Globally the problem remains the same, with limited options for treatment of POP available to women [11, 12]. Currently in the UK, numbers of specialist women’s health physiotherapists within healthcare settings is variable and limited, with only 800 currently registered with the Pelvic, Obstetric and Gynaecological Physiotherapists (POGP) - a professional network group of the Charter of Physiotherapists. Based on data from the 2011 UK census and taking a conservative estimate of women with POP, there are approximately 2600 women with symptoms of POP for each specialist physiotherapist. It is therefore very unlikely that these women will be able to access PFMT unless different service configurations or formats, including delivery by different healthcare professionals, become available. Therefore, in order to enhance service capacity and increase availability and choice of PFMT for women, research into different models of service delivery is required.

Implementation is now well known to be a complex and challenging process [13–15]. As a first step towards implementation, it is important to know whether PFMT can feasibly be delivered within the NHS (and eventually beyond the UK setting) using different staff skill mixes and whether outcomes are comparable to those achieved under trial conditions. In addition, it is vital to understand what specific barriers and facilitators to delivering PFMT may operate in different contexts, what strategies could be used for successful implementation and what might be the underlying mechanisms of their action.

With these gaps in knowledge, the PROPEL project was designed to maximise the delivery of effective PFMT for women with prolapse through the study of its implementation in five diverse real-world settings [8]. This involved developing different service delivery models, such as using different staff skill mixes to deliver PFMT, with the format and setting of delivery being determined by local clinical and managerial teams. The project team consisted of consultant gynaecologists, specialist physiotherapists, academic experts and women with POP. It was overseen by an independent steering committee comprised of specialist physiotherapists, nurses and women with POP. We conducted a realist, outcome and economic evaluation of the implementation of PFMT using different delivery models (see Maxwell et al. 2017 [8] for full methods). No changes or deviations were made from the published protocol. The findings from the outcome and economic evaluation are reported in [16] (Berry et al. PROPEL: Patient reported outcomes study of pelvic floor muscle training for women with pelvic organ prolapse. In preparation) respectively. This paper reports on the findings from the realist evaluation (RE) of this implementation, which aimed:

1. To understand the barriers and facilitators to implementing PFMT across varying NHS settings from managerial, delivery staff, and women’s perspectives and experiences, and to develop different models of delivery in response to these.
2. To contribute to knowledge of how and why implementation processes are successful (or not) through exploring what works, for whom and in what circumstances.

## Methods

### Theoretical frameworks

The overall PROPEL study was informed by two theoretical frameworks from implementation science theory:

Realist Evaluation [17] was used to understand how the intervention was implemented in different study sites, what contextual factors influenced its implementation and what ‘mechanisms of action’ lead to successful (or unsuccessful) delivery and outcomes. Realist evaluation contends that it is not interventions that work, rather it is the people involved in interventions who make them work. It is people’s reasoning and capacity in response to the intervention elements which represent the real ‘mechanisms of action’. These mechanisms of action are however contingent on the social context in which people work. Certain contexts enable people to act while others place limits on their behaviour [18]. Realist evaluation thus seeks to explain the complex relationship between the mechanisms (M) activated by the intervention, the context (C) that influences their workings and the intended and unintended outcomes (O) they produce [19].

This study aimed to implement a complex intervention (the delivery of PFMT using different staff skill mixes) in complex NHS systems (consisting of a number of actors, varying resources, diverse geographical locations and service configurations) through a complex implementation process (requiring actions and decisions from people in multiple roles at different levels). Given the interplay of multiple factors operating in different personal or organisational contexts with different priorities and goals, realist evaluation provided an appropriate framework and methodology to explore and explain the implementation of PFMT.

RE-AIM [20] was used to determine the translational quality and overall public health impact of the intervention using five key dimensions. Reach referred to the extent to which the target population was touched by the intervention. Effectiveness referred to the impact of intervention on women’s prolapse and quality of life outcomes. Adoption referred to the willingness by target settings, institutions and staff to implement, support and embed the intervention into their routine practice. Implementation referred to the fidelity and consistency of intervention delivery as intended. Maintenance referred to the extent to which an intervention becomes institutionalised or part of the organisational practices/policies. These dimensions were assessed using a range of qualitative and quantitative data gathered across the realist, outcome and economic evaluations.

### Setting

The study took place in five diverse NHS sites across the UK, two in Scotland and three in England, within either specialist pelvic floor, musculoskeletal physiotherapy or women’s health services. The sites reflected a mix of urban/rural locations, previous involvement/non-involvement in the original POPPY trial, and current differences in service delivery models. A brief description of these sites can be found in Table 1. Sites A, B and C were involved from the conception of the study and took part in all phases of the realist evaluation. Two sites, D and E, were added nearly 12 months after the commencement of the study to help achieve women’s recruitment targets for the outcome evaluation. As the data collection for the first phase of realist evaluation had been completed by this time in sites A, B and C, the two new sites (D and E) were only involved in the second phase of the RE.

**Table 1:** Context of services and care in study sites

Site	Service context	Service model adopted	Skill mix trained
A	Urban, POPPY site	No change. Existing primary and secondary care provision of specialist physiotherapy. Referrals triaged.	Specialist physiotherapists (existing team) (2 x band 7, 5 x band 6 women’s health physiotherapists)
Care context	• Service proudly described as gold-standard care – adequate numbers of highly trained staff, good working relationships and communication flow, team approach to practice, well resourced. • Improvements seen to be needed in raising awareness among GPs to enable direct referrals, improving waiting times, referral pathways and follow-up care.		
B	Rural	PROPEL PFMT training provided to a variety of clinicians over a large geographical area. Including clinicians with special interest, district nurses, continence nurses and physiotherapists. PROPEL women triaged by specialist physiotherapist prior to referral into the PROPEL service. Community and secondary care based.	2 x Musculoskeletal (MSK) physiotherapists band 6 1 x General physiotherapist band 6 2 x District nurses 1 x Lead nurse specialist in continence band 6 2 x Urogynaecology nurses
Care context	• Incontinence service worked closely with physiotherapy, but seen as ‘pad provision’ service, needing to become more holistic and proactive in assessment and treatment • Staff shortages prevalent – patients and staff needing to travel long distances • High levels of motivation among staff, many with special interest in women’s health. Service had history of training MSK physiotherapists in PFMT delivery. Support from management was strong.		
C	Urban	New provision of PFMT delivery developed for PROPEL based in secondary care. Consultant triaged and referred into PROPEL service provided by urogynacology nurses	3 x Urogynaecology nurses trained, 2 took part in PROPEL
Care context	• Perceived to have lack of co-ordination between primary and secondary care services with regards to prolapse and incontinence • Perceived need for service design and some level of enthusiasm about PROPEL among acute and community nurses, management and some consultants.		
D	Urban	Community healthcare setting. Current PFMT service delivered by small number of specialist physiotherapists. 4 clinicians to deliver PROPEL service in a community healthcare setting	4 x MSK physiotherapists (1 x band 5, 2 x band 6 and 1 x band 7)
Care context	*No phase 1 data available*		
E	Urban	Current PFMT service delivered by small number of specialist physiotherapists. 4 trained clinicians to deliver PROPEL service in a community healthcare setting	2 x Urogynaecology nurses 2 x Physiotherapists (1 x band 5, 1 x band 6)
Care context	*No phase 1 data available*		

### The PROPEL implementation

PROPEL project aimed to maximise the delivery of PFMT to women with prolapse. The precise format and setting of PFMT delivery was expected to be determined locally by individual sites during the service planning stage [8]. The sites were asked to develop their bespoke models of service delivery through local stakeholder engagement i.e. by making decisions on settings for service delivery (community, primary, secondary care), appropriate skill mixes for delivery (specialist physiotherapists, women’s health nurses, and junior, band 5 physiotherapists) and number of sessions involved in PFMT. Once potential staff were identified to be trained to deliver PFMT during service planning stage, they attended a one day training session held within their site, developed specifically for this study in conjunction with POGP. It was delivered by two POGP-registered specialist physiotherapist trainers to a maximum of 5 new staff/site per session. Training manuals were produced and provided to the participants. Further information about the training can be found in the project report [16].

### Design

Informed by the realist framework, the evaluation was conducted in three broad phases, using a longitudinal, multiple-case study design [21]. The five study sites were considered as ‘cases’, which were defined at the level of the ‘NHS trust’ in England and ‘NHS health board’ in Scotland as these represent the units through which health services are organised, governed and delivered in local areas. Defining the ‘cases’ at the level of these broad units helped ensure that the influence of contextual conditions at various levels (i.e. from financial, organisational and managerial to clinician, practice and patient level) was encompassed in the evaluation. The study was approved by the NHS Wales Research Ethics Committee 7, REC number 15/WA/0427.

### Data collection

The three phases of the realist evaluation aimed to identify, test and refine a theory explaining how and why the PROPEL intervention worked (or not). It involved data collection at 4 time points over an 18 month implementation period. The methods used in each phase are outlined below.

### Phase 1 – identifying folk theories of change

Phase 1 took place during the planning stages of the intervention through two rounds of data collection aiming to a) track local decisions on what to implement, how and why and b) elicit folk theories of change from key stakeholders about the likely outcomes of the intervention, possible mechanisms of action and potential contextual influences.

#### Round 1 and 2: development and operationalisation of the service delivery models

##### Focus groups with women

The research team conducted focus groups with women receiving care for prolapse in each study site in round 1 exploring their experiences of local services and care and visions for a responsive and woman-centred service. Summaries of data from these focus groups were fed back to each site to provide service-user input to service delivery decisions. Findings from the focus groups are published elsewhere [6].

##### Service planning meetings

The liaison specialist physiotherapist in each site identified and invited local service managers, clinical leads, consultants, and other relevant staff groups to attend a series of service planning meetings. First meeting, convened in round 1, considered the evidence base and potential benefit of PFMT, current service provision, local capacity issues and initial options for service delivery models with the available or extended staff pool. Second meeting, convened in round 2, selected and finalised the service delivery model to be implemented, planned its operationalisation and identified staff groups for training in PFMT delivery. Both meetings were audio-recorded to track the decisions being made as well as the folk theories around potential contexts, mechanisms and outcomes (CMOs). The decisions were finalised over two planning meetings in sites A and B and three meetings in site C.

##### Semi-structured interviews

Individual semi-structured interviews were conducted with a number of stakeholders in each site in two rounds. Service leads/managers and senior practitioners (Urogynaecology consultants/senior nurses or allied health professionals) who were likely to be key decision makers were identified from the service planning meeting attendees, with further interviewees identified through snow-ball sampling. Round 1 explored the contextual details about the site, anticipated barriers and facilitators to implementation, potential mechanisms in terms of staff’s attitudes, and anticipated outcomes. Round 2 explored views and reactions of various staff towards the new service model, its operationalisation, potential barriers and facilitators to implementation, and intended and unintended outcomes. In round 2, additional interviews were also conducted with staff being asked to deliver PFMT under the new service model to explore their views on the new service model, expectations of training and new role, concerns and anticipated problems. The exception to this was site A where these staff groups were only interviewed once in round 2 as this site decided not to implement any changes to their existing service models. This may have been influenced by their involvement in the original POPPY trial and this site served as a real world case study of PFMT delivered by specialists (outside of trial conditions) as well as a ‘gold standard’ model to compare outcomes from other delivery models. The interviews in this site focused more on how the current service was organised, delivered and working, whether adaptations had occurred, what worked well and why, and what needed improvement. See supplementary file 1 for topic guides.

#### Round 1 and round 2 data analysis

Data from round 1 and 2 interviews and service planning meetings were transcribed verbatim and analysed using the thematic framework approach [22] adapted for use in realist evaluations [23]. Data analysis was conducted by three members of the research team (PA, JW, IU) in parallel with data collection. A coding frame was developed in round 1 using data from two transcripts, summaries of the first service planning meeting from sites B and C and the C-M-O framework. Following familiarisation, codes were assigned to data segments reflecting the meaning contained in segments in relation to main topics covered in interviews. These codes were grouped together under higher order themes and classified as describing either a context, a mechanism or an outcome (see Table 2 for descriptions of these categories). This initial coding framework was systematically applied to all the transcripts from round 1 and 2 with new codes added as emerging from subsequent data. Once all the data had been coded, the coding framework was used to identify linked patterns of context-mechanisms-outcomes and generate initial CMO configurations. Theories of change were first identified for each site and then compared across sites to look for CMO patterns that cut across the site boundaries.

**Table 2:** Classification of codes according to key realist evaluation concepts

RE concepts	Classification of codes
Context	Codes describing any pre-existing factors outside the control of intervention designers such as social or service structures, enabling or disabling conditions, resources, relationships, cultures, staff/service capacities and motivations. Codes describing something that developed/emerged/changed during the intervention but was unrelated/not attributed to the intervention itself.
Mechanism	Codes suggesting a change in people’s minds and actions (reasoning, feelings, behaviours, judgements, decisions and attitudes at individual, interpersonal, social and organisational levels) in response to the changes introduced by the implementation as well as those described as interim outcomes of the intervention
Outcome	Codes describing the intended and unintended consequences of the intervention at the level of women, staff or services (whether higher level outcomes or indicators of higher level outcomes)

### Phase 2 – testing the folk theories of change

The initial folk theories of change were tested, in two further rounds, by collecting data on contexts, mechanisms and outcomes at operational level in each site to explore how the intervention was implemented and worked in different areas.

#### Round 3 and round 4: delivering and reviewing the models

##### Semi-structured interviews

Round 3 interviews took place ‘during’ the implementation stages once staff had begun to deliver PFMT to women under the new service model and explored how the new service model was operating and any problems that had arisen during implementation. Round 4 took place after the intervention period had ended, as dictated by the achievement of site-specific recruitment and treatment target, and explored whether the implementation was perceived to be successful, whether/how it worked, what lessons were learnt and the plans for continuation of PFMT delivery locally. See supplementary file 1 for topic guides. In both rounds, interviews were conducted via telephone with service leads/managers, consultants/senior nurses/allied health professionals (AHPs)/ general practitioners (GPs), staff delivering PFMT, and women receiving PFMT from the newly trained staff. Where possible, each successive round of data collection included the same participants as previous rounds. In case of staff changes, unavailability or withdrawals from study, new participants were added through snowballing.

#### Round 3 and 4 data analysis

Data from round 3 and 4 were analysed by three members of the team (PA, JW, TA) using the framework approach similar to that described in phase 1. Once all the data had been coded, data from each transcript were summarised and tabulated using a framework consisting of rows indicating a data source and columns indicating the contexts, mechanisms and outcomes. For each site, the data summaries were compared across the participants to provide a ‘story of implementation’ by understanding the outcomes of implementation in each site, their underlying mechanisms of action and the contextual factors triggering those mechanisms. The next phase of analysis focussed on ‘testing’ the initial theories of change identified in phase 1 for their adequacy in explaining the observed patterns of CMOs. This involved explicitly comparing the observed CMO patterns (how and why the intervention actually worked or not) with hypothesised CMO patterns (how and why it was expected to work). The analytical process was outcome-led; i.e. we began with the groups of outcomes that were anticipated in phase 1 folk theories to result from the intervention and looked for evidence in phase 2 data on how much or how well those outcomes were achieved in each site. We also mapped these outcomes onto the elements of the RE-AIM framework, except for ‘maintenance’. Maintenance of the service delivery models developed during PROPEL in the longer term could not be explored as the sites were not followed up beyond the project period. We then sought to explain the observed outcomes in each site by looking for the possible linked mechanisms and contextual factors that appeared to trigger those mechanisms. These constituted the site-specific CMO configurations.

### Phase 3 – developing middle-range theories of change

Once the site-specific CMOs were developed explaining when, why and for whom certain outcomes were achieved (or not), cross-case comparisons were performed to refine the CMOs and develop middle-range theories about the intervention. For each outcome, we compared and contrasted the CMO models emerging from all the sites. The analysis was carried out a higher level of abstraction, transcending the individual sites. The CMOs were refined by identifying the facilitating or impeding contextual factors that were common across the sites and re-examining the linked mechanisms in relation to each outcome. This meant that a particular CMO was now able to explain the workings of the intervention in more than one site where the specific contextual factors were present.

Table 3 presents the number and type of data sources collected in rounds 1 to 4 during phase 1 and 2 of the realist evaluation.

**Table 3:** Number of participants^a^ in the realist evaluation (By NHS site)

Phases of RE	Data collection rounds	Participants in service Planning Meetings (SPMs)	Managers/Service Leads	Senior Clinicians	Staff delivering PFMT	Women
**Phase 1**	**Round 1**	Total = 12	Total = 5	Total = 2		Total = 21
		B = 4 (1 SPM) C = 8 (1 SPM)	A = No interviews B = 3 C = 2	A = No interviews B = 1 C = 1	No interviews in this round	Focus groups = 17 (A = 1, B = 2) Interviews = 4 (All in site C)
	**Round 2**	Total = 26	Total = 6	Total = 3	Total = 11	
		A = 11 (1 SPM) B = 7 (1 SPM) C = 4 + 4 (2 SPMs)	A = 3 B = 2 C = 1	A = 1 B = 1 C = 1	A = 4 B = 5 C = 2	No interviews in this round
**Phase 2**	**Round 3**	N/A	Total = 10	Total = 4	Total = 10	Total = 18
			A = 2 B = 1 C = 4 D = 3	A = 1 B = 1 C = 2	A = No interviews B = 7 (incl. 2 exit interviews) C = 2 D = 1	A = 7 B = 8 C = 3
	**Round 4**	N/A	Total = 5	Total = 2	Total = 18	Total = 15
			A = 1 B = 1 C = 2 E = 1	A = 1 C = 1	A = 7 B = 8 C = 1 D = 1 E = 1	A = 6 B = 6 C = 3

^a^Where possible, each successive round of data collection included the same participants as previous rounds. In case of staff changes, unavailability or withdrawals from study, new participants were added through snowballing

## Results

### Phase 1 – folk theories of change: how was the intervention expected to work?

The PROPEL intervention introduced an opportunity to deliver PFMT using different staff-skill mixes to a wider population of women with prolapse than that currently reached by specialist physiotherapy services. This additionally provided an opportunity to reconfigure the local service and referral pathways, training in PFMT delivery to identified skill-mixes and resources to support the new model of service delivery such as funding to specialist physiotherapists for providing support, part funding to newly trained staff and direct funds to health boards/NHS trusts. Across the sites, the intervention was expected to impact on three key sets of outcomes: a) a public health impact by way of widening the reach and accessibility of PFMT to the target group in local areas; b) impact on women’s health by way of improvements in prolapse symptoms and quality of life, reduction in surgeries; and c) impact on services by way of shortened waiting lists for PFMT and reduction in specialist workload so that their resources can be focussed on more complex cases.

**Fig. 1:**
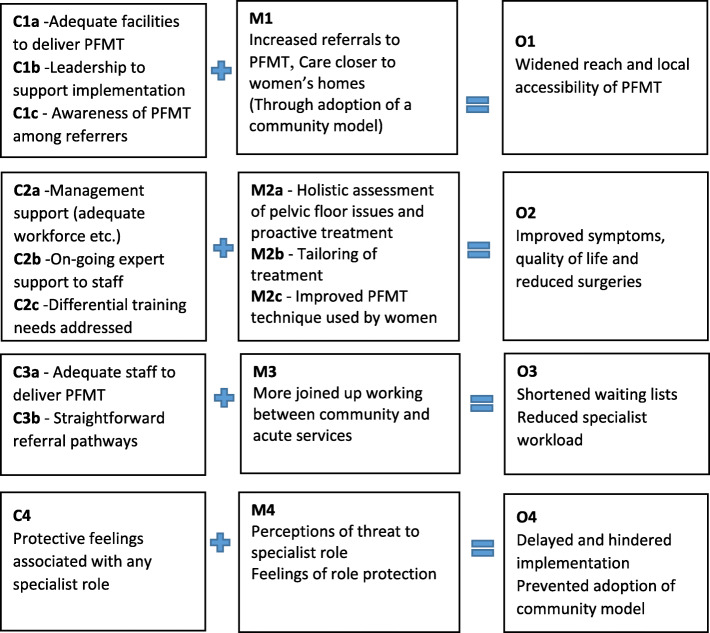
Initial CMO configurations developed in Phase 1

The context and organisation of prolapse care varied significantly among the three sites. Several contextual factors were identified in round 1 and 2 data that seemed likely to influence the implementation of the intervention, which in turn would impact on the achievement of anticipated outcomes. Table 1 describes the context of care in the study sites. Four sets of CMO configurations (Fig. 1) were identified from the data containing folk theories around how each of the intended outcomes would be brought about and what may facilitate or impede these processes. The data also revealed an unintended outcome that was expected to affect implementation (see CMO 4 below).

#### CMO configuration 1 – widening the reach of PFMT through increased local provision of care

It was anticipated that using different staff skill mixes to deliver PFMT (I) would widen the reach and accessibility of PFMT in local areas (O1) because it would increase the number of referrals to PFMT and the provision of PFMT in the community, closer to women’s homes (M1) through adoption of a community model of service delivery. This mechanism was dependent on whether adequate facilities (e.g. private rooms in clinics) were available to carry out assessments and deliver PFMT (C1a), whether there was strong leadership in services to support the work (C1b) and whether GPs and other potential referrers were aware of PFMT and new referral options (C1c). In sites B and C, there were concerns that lack of appropriate facilities may prove challenging to successful implementation.

#### CMO configuration 2 – improving women’s health outcomes through holistic and proactive care

The intervention was expected to improve women’s symptoms, quality of life and reduce the need for surgeries (O2) by enabling the newly trained staff to perform more holistic assessment of urinary incontinence and pelvic health issues and provide proactive treatment in the form of PFMT (M2a), by enabling them to tailor the treatment and advice based on accurate assessments (M2b) and by informing women of the correct PFMT technique (M2c). Whether these mechanisms would materialise or not depended on the level of support from the service leads/management in terms of ensuring dedicated time to deliver PFMT, sufficient workforce to deliver existing and new service and manageable workloads (C2a), availability of on-going support by specialist physiotherapy staff to newly trained staff (C2b) and acknowledgement of differential training needs of different staff (e.g. nurses vs. physiotherapists) (C2c). In sites B and C, the staff shortages and existing staff roles and workload were expected to act as barriers to successful implementation. Concerns were also expressed about the impact on workload if the demand increased due to greater awareness among GPs and women.

#### CMO configuration 3 – improving service organisation through joined-up working

The intervention was expected to improve service delivery by reducing specialist workload and shortened waiting lists (O3). This was expected to result from more joined-up working between physiotherapy and nursing teams in the community and acute settings (M3). This was possible only if there were adequate number of staff trained to deliver PFMT (C3a) and the referral pathways were straightforward (C3b). There were some concerns that pressure to reduce the waiting lists may actually lead to inappropriate referrals to newly trained staff, which in turn would increase rather than decrease the waiting times and specialist workload.

#### CMO configuration 4 – implementation difficulties due to role protection issues

In addition to the intended mechanisms and outcomes, the interviewees anticipated a fourth, unintended mechanism that had already started to unfold and impact implementation processes during the initial stages. With the intervention bringing the prospect of training other staff, particularly those of a lower banding/grade, this triggered perceptions of threat to the role of practitioners specialising in pelvic health or PFMT and activated feelings of role protection among these staff (M4). A reluctance was sensed among the specialist staff, particularly in sites A and C, to train nurses of a lower banding to perform the higher skilled tasks (such as deliver PFMT which has been a specialist job), which was felt to be causing a disservice to the specialist profession. This reluctance was also observed in the form of some ‘hostility’ at the service planning meetings. The feelings of role protection were said to be always present (C4), but these were observed to resurface and intensify as a result of the PROPEL intervention. This mechanism of ‘role protection’ hindered and delayed the process of implementation in sites A and C and prevented adoption of a community model of PFMT delivery involving band 5 community nurses (O4). Site A continued their service through hospital and community-based specialist physiotherapists without making any changes to the service model, whereas site C had to abandon the plans for training nurses or physiotherapists in the community and adopted a hospital based model involving hospital based Urogynaecology nurses.

### Phase 2 – testing the folk theories

Findings from this phase consist of site-specific CMOs (presented in Additional file 2) reporting the outcomes of the implementation that were observed in each site and attempt to explain how and why these outcomes were achieved, for whom and in what contextual conditions. The outcomes include those that were expected in the initial theories of change as well as those unintended and unanticipated.

### Phase 3 – refined intervention theory: how did PROPEL intervention work, for whom and in what contexts

**Fig. 2:**
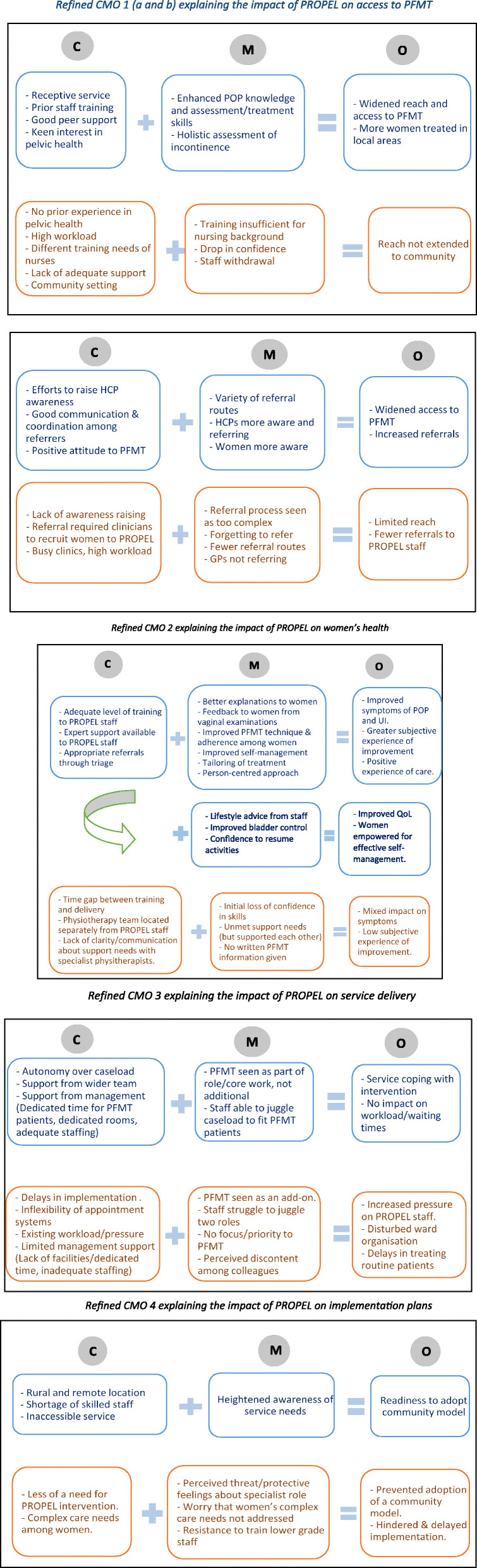
Refined CMOs developed in Phase 3

Refined CMO configurations are presented in Fig. 2. Quotes from participant interviews illustrating the different refined CMOs can be found in Table 4.

**Table 4:** Quotes illustrative of refined CMOs

Facilitators	Barriers
**Refined CMO 1a explaining the impact of PROPEL on access to PFMT**	
‘I think although I did have the advantage of being a physio over perhaps my nursing colleagues, who I know felt a bit overwhelmed with the whole process of doing it, so, yeah, I felt sort of in the middle where obviously, at the time, I hadn’t had much experience, that was the first time I’d actually treated anybody with a prolapse. But, yeah, I’ve gone on to treat more and I think the training was great, it was good, but what helped me more was the confidence of having worked alongside a specialist. H011 (MSK Physiotherapist with special interest in women’s health) [Rd 4 Intv 2]	‘I do remember feeling very uncomfortable [at the training session]. I don’t know why. I just think...I think I felt uncomfortable mainly because anybody else that was there probably had some kind of experience doing [internal] examinations and things […] but I had never, ever, done anything like that.’ H006 (Community Nurse & Link Nurse for continence) [Rd 3 Intv] 2 [Withdrew from study]
**Refined CMO 1b explaining the impact of PROPEL on access to PFMT**	
‘Certainly the GPs are more aware and I suppose where they used to just send patients to me who just wanted incontinence pads, well now they send them for other continence issues as well, kind of thing. But they send them more for treatment rather than for just management, if you know what I mean.’ H007 (District Nurse, including Continence Link Nurse) [Rd 3 Intv 2]	‘I’ve noticed that my caseload has been particularly quiet this year. I’m not quite sure of it definitely but I’m thinking {PFMT service] it’s maybe just not known as much. I’ve sent out emails. I sent one out to the GPs about a year ago to try and flag up that I was participating in the study and just to try and get some referrals coming in. The ones I’ve had since I have noticed that some of them have been referred straight to gynaecologists, and gynaecologists have referred them back to physio. So, I don’t know if there’s a bit of GPs just not sure of the service.’ H020 (Community Physiotherapist, MSK & women’s health) [Rd 4 Intv 2]
**Refined CMO 2 explaining the impact of PROPEL on women’s health**	
‘Oh, I’d be 100 % a positive experience, and I’m glad that I had that experience. I’m not glad that I’ve got a prolapse, but I’m glad I’ve had the experience of discussing it with the physios, and being shown how to do the exercises probably better than I would have done them without any intervention by physios. So my experience is a very positive one.’ H019 [Rd 4 Intv 2] *‘*It was excellent, because the benefits I’ve reaped from it. […*]* the prolapse is not cured 100 %. But I have, I feel I have quality of life back, I have control over it.’ G012 [Rd 4 Intv 2]	‘We’d forgotten the training by the time we started to […] see the patients, and that’s why we started to [deliver PFMT] together […] Because we felt more confident the two of us doing it […]. L015 (Gynaecology ward nurse) [Rd 3 Intv 1] ‘But we were concerned that, Were we doing it right?, Were we good enough?. […] We were a little bit concerned.’ L015 (Gynaecology ward Nurse) [Rd 3 Intv 1]
**Refined CMO 3 explaining the impact of PROPEL on service delivery**	
‘I think it fits in really well with my workload; it doesn’t impact it at all.’ Med 005 (MSK Physiotherapist) [Rd 4 Intv 1] ‘I think in terms of a clinical research team, the admin team, the physios, everybody is proactive about what they are doing and also nudge each other in doing what they are doing. […] So, kind of, being proactive about what you are doing and having the general set-up and pathways, I think leads to that, and good managers who actually do allow you to be available for such kind of research that is going to enhance the service, I think is what’s needed. […] So yes, a good manager and a good team. M003 (Manager/Service Lead, Physiotherapy team) [Rd 3 Intv 1]	‘Well, we both work as staff nurses on the gynae ward, and then we’ve been trying to sort of carry out the PROPEL study within that role, which, in itself, we feel has been quite difficult. Because you’re sort of on the ward one minute and you’re in charge of the ward, or in charge of the patients, and then we’re having to switch off of that and go over and do a PROPEL’s lady, which can be quite difficult, can’t it?’ L015 (Gynaecology ward nurses) [Rd 3 Intv 1]
**Refined CMO 4 explaining the impact of PROPEL on implementation plans**	
‘Well, I suppose I’ve not really spoken to...apart from with people who are the physios and nurses. And I think their response to that is quite positive about it being...because we live in a remote and rural area I think we’re far more open to services being delivered more widely because patients have such long journeys to travel to get treatment. So as you know we have some specialist physios up here who have already trained people throughout NHS Highland to be able to deliver it in other remote and rural areas, because otherwise we were only seeing just such a small number of people that actually were needing to be seen. So I feel that that’s why we’re thinking this is quite good if we can get some nurses on board and trained up as well’. H (Senior AHP 002), [Rd 1, Intv 1] ‘Well, particularly for places like [place name] where we don’t have a specialist physio I think it would be useful, and because I know also the physios are understaffed, so I don’t think it matters. As long as we’ve got somebody who’s got the training, whether that’s the physio or a community nurse, as long as it’s available to the patients then I think that would be beneficial’. H (District nurse 002), [Rd 2, Intv 1]	‘Well, the obvious one (barrier) appears to be role protection. It seems to me it’s a bit unfair to say that but I got the distinct impression that there was reluctance to train people of a lower deemed banding or skill mix to do something that was more skilled’ L01 (Service manager) [Rd 1, Intv 1] And, you know, again, for someone who’s just had the basic training, yes, they might be able to treat someone who’s very basic … you know, like a stress incontinence patient, or someone who’s very, you know, straightforward. But not, you don’t get many people like that, from my experience. Most of my patients with prolapse symptoms will have, you know, maybe overactive bladder symptoms, or stress incontinence, they might have sexual dysfunction. So you might find that there’s a lot more there, that if someone has only done a brief training course, that that’s way more advanced for them, you know, and having bowel incontinence issues. And again, it’s, I think it’s difficult to train someone up, unless you’re a specialist physio within, you know, pelvic, obstetric, and gynaecology physiotherapy. G006 (Specialist physiotherapist) [Rd 4 Intv 2]

#### Reach – impact on access to PFMT

The extent to which the ‘reach’ of PFMT to target population was widened following PROPEL varied across the study sites, depending on the presence or absence of two key contextual factors: a) the receptiveness of the clinical setting and b) the level of awareness of PFMT among potential referrers.

The access to PFMT widened in areas where the service was receptive to PROPEL – staff and management had keen interest in pelvic and women’s health and had already started providing additional training to other staff to increase capacity and there were good peer support networks in place. This was mainly observed in the case of MSK/general physiotherapists and some nurses. In these contexts, PROPEL training enhanced staff’s knowledge of prolapse and their skills and confidence in assessment and treatment. Transition to undertaking specialised assessments and PFMT was easier and faster for these skill-mixes due to their basic physiotherapy training or CPD training in pelvic health assessment and management. Nurses caring for women with urinary incontinence and other pelvic health issues extended their knowledge and skills beyond POP. Their enhanced knowledge led them to become more holistic in the assessment of pelvic issues and proactive in providing treatment.

However, different sets of mechanisms and outcomes were observed in nurses in community setting who had no prior experience or training in pelvic health. A key underlying mechanism was low levels of adoption by community nursing staff which was reflected in their withdrawal from the study. For a small number of professionals, the training was felt to be inadequate to address their knowledge and skill gap, as they did not have the in-depth understanding of physiology that physiotherapy training provided. This lowered confidence and led to withdrawal from the study. For the advanced nurse practitioner for continence, the existing workload and a mismatch of expectations from their role led to withdrawal. Community setting was considered inappropriate for prolapse assessment and PFMT delivery as these were not feasible in people’s homes or care homes and PFMT was deemed unsuitable for older people and those with dementia, both of which comprised the main aspect of their role. As a result, contrary to original intentions, PFMT failed to be extended to community settings (Refined CMO 1a).

Another reason for the difference in PFMT reach was the level of awareness of PFMT services among professionals who could refer women to PFMT. In contexts, where the teams made efforts to raise other HCP’s awareness of PFMT, there was good communication and coordination among referrers and there were favourable attitudes to PFMT, access to care was widened. This was because there were a variety of direct referral routes available, GPs and secondary care consultants were referring patients to these services and women being more aware were spreading the word in community. In contrast, the referrals were restricted in areas where not much effort was made to raise awareness among potential referrers which meant their referral patterns remained unchanged. Referrals were also restricted because the consultants remained distant and disengaged in PROPEL and other possible referral routes were not exploited either. Referral required clinicians to recruit women to the PROPEL study, which included additional tasks such as determining women’s eligibility for study through an internal vaginal examination, giving study information, taking consent and performing baseline assessments. This was difficult to do in busy, short-staffed clinics. The process of referral to PROPEL was seen as more complex than routine and was hence was often overlooked (Refined CMO 1b).

#### Effectiveness – impact on symptoms, quality of life and care experience

Effectiveness in the context of PROPEL study was concerned with the impact of the PFMT delivery by different staff skill mixes on women’s symptoms, quality of life and experience of care. The outcome evaluation [16] revealed that there was a significant improvement in outcomes after the intervention than before, but no significant differences in outcomes across the five sites and across different delivering staff and across different service models. This suggested that the outcomes were comparable regardless of the study site, by whom and through which model. However, the staff and women’s perceptions of their outcomes were found to differ from the actual clinical evidence of improvement and across different sites due to differences in implementation processes. The refined CMOs below explain these differences in perceived outcomes.

Many women and staff reported improvements in prolapse symptoms and incontinence. In some cases, the experience of improvement was greater than clinical evidence. When improvements in symptoms were reported, the underlying mechanisms seemed to be women receiving better explanations of prolapse and the role of PFMT from staff delivering this. These included feedback on their performance from assessments, tailored and structured PFMT routine, advice on correct PFMT technique, and techniques for exercising regularly, all of which improved women’s adherence to PFMT. The reported improvements in quality of life resulted from staff enabling women’s self-management of their symptoms by offering tips and advice on lifestyle, which helped improve women’s bladder control and increase their confidence to resume previous activities. There was an overall person-centred approach as staff were seen as approachable, motivational, dedicating enough time, reducing women’s embarrassment, and making them comfortable during appointments. This resulted in a positive and satisfactory experience of care. All the above mechanisms were triggered in contexts where the staff were adequately trained and confident, had expert support available throughout, and when the referrals were appropriate for the level of care they provided.

In contrast, certain contextual factors failed to trigger the mechanisms which led to perceptions of improvement in symptoms, quality of life and care. This was particularly the case where the staff delivering were located in an acute ward setting. Staff and women reported fewer improvements in symptoms, despite the clinical improvement observed in the PROMS study indicating otherwise. A number of factors present in these contexts seemed to trigger certain mechanisms that led to less experience of improvement. First, there was a time gap between training and PFMT delivery which led to an initial loss of confidence among staff. Second, the specialist physiotherapy team was located separately from the acute ward where PROPEL nurses were located and there was a lack of clarity and communication on both sides about the staff support needs. This triggered another mechanism whereby staff supported each other by doing joint clinics helping with care as well as confidence (Refined CMO 2).

#### Adoption – impact on service delivery

Adoption refers to willingness of institutions and staff to implement an intervention and support its adoption into routine practice. In this study, one of the indicators of adoption - the uptake and continued participation (or drop-out) by staff – was already reflected in the mechanism leading to wider (or restricted) reach of PFMT to target populations. Another indicator of the extent to which the intervention was adopted into practice is the impact of PROPEL intervention on the services in which PFMT was delivered by other skill mixes. Favourable impact on service outcomes would indicate higher levels of adoption, while unfavourable impact would indicate lower levels of adoption. It was hypothesised that the intervention would reduce waiting times and specialist workloads by creating more joined up working between acute and community services. While there was little evidence for this mechanism in the theory-testing phase, a number of other outcomes and their underlying mechanisms ensued, for instance, impact on workloads and pressures on delivering staff as well as their professional colleagues, organisation and functioning of the services and perceived support from wider team.

In some areas the intervention was better integrated and adopted into the routine service than others and had little disruptive impact on workload, service organisation or waiting times. This was particularly in contexts where staff, mainly physiotherapists and nurses in some settings, had some degree of autonomy over managing their appointments and caseloads and the wider team supported them in PFMT delivery. They were also supported by their service management by allowing dedicated time for PFMT delivery, making rooms available for assessment and treatment, and providing adequate staffing resources. This triggered the mechanism wherein PFMT was seen as part of their core role, rather than as additional.

Conversely service disruption was reported in some areas in the form of disturbed ward organisation, delays in treating routine patients, and increased pressure on staff, particularly in case of nurses in acute wards and community settings. This was because PFMT delivery in these settings was seen as additional work, not made a priority or given due recognition. Staff had to juggle two roles and required additional administrative support. Colleagues were perceived to be discontent about others’ involvement in PFMT as they often had to provide cover for them. This resulted in doubts about the extent to which such an intervention may be supported by the wider team. Several unfavourable factors in the context played a role in triggering these mechanisms. First, delays in implementation and inflexibility of the patient booking systems meant that appointment slots for PROPEL patients remained unfilled during early phases and led to delays in treatments of routine patients as appointments could not be allocated to them. Second, existing workload pressures in the service areas along with lack of dedicated time for PFMT, lack of appropriate facilities and inadequate staffing led to additional pressure (Refined CMO 3).

#### Implementation – impact on implementation plans

Implementation refers to the extent to which the PROPEL intervention was delivered/implemented as intended by the services. During the implementation phase, the services in sites A, B and C considered and debated various service configurations and potential skill mixes, with the overall intention of widening access to PFMT in local communities. Whether this intention was accomplished or not was determined by the activation of the ‘role protection’ mechanism triggered in certain contextual conditions.

In remote and rural contexts there were significant staff shortages and the service was inaccessible in many areas. There was heightened awareness of the service needs among the implementers, at least at implementation stage. This led to an increased readiness to adopt a community model and train nursing staff in the community. However, in other contexts where there was adequate specialist capacity, the implementers were less convinced of the need for the PROPEL intervention. This triggered the feelings of threat to their role and protective feelings about this. These staff were also concerned that training lower grade staff may not address women’s complex care needs adequately and would impact negatively on standard of care. These feelings resulted in preventing the adoption of a community model, resistance to train lower grade nurses and significant delays in implementation (Refined CMO 4).

## Discussion

PROPEL aimed to maximise the delivery of PFMT to women with pelvic organ prolapse by implementing different models of service delivery across five NHS sites and models. The service delivery models were designed and implemented by the sites locally through active stakeholder engagement and involved PFMT delivery by different skill mixes including specialist, MSK and other physiotherapists and/or nurses. The comparison of women’s reported symptoms following care received under these different models (reported in [16]) showed that the PROPEL intervention ‘worked overall’ as symptoms improved regardless of the service model and skill mix through which PFMT was delivered. The realist evaluation allowed us to study how the different models were developed and implemented by sites and how and why their implementation worked (or not) in diverse contexts differing in their geographical locations, service organisation, type of service model used, the skill-mix trained, and availability of support structures and resources. Our findings have particular relevance to urogynaecology services aiming to increase PFMT service provision but also have important implications for other services and health systems implementing evidence-based interventions related to role expansion. Below we summarise and explain our findings with reference to theoretical and empirical literature related to professional identities and role expansion.

First, we found that a heightened awareness of the need for services among staff and management was a mechanism for change, particularly in areas where there was a shortage of skilled staff. It enhanced the staff’s readiness to extend the reach of PFMT services in the community setting, train different staff types and initiate ‘workarounds’ to enable triage and referrals to the new services. In contrast, the service with the most established model of specialist physiotherapist-delivered PFMT was the most resistant to trying alternative models regardless of need as this activated role protection feelings and concerns about compromising care quality. This finding can be explained using the concept of professional identity threat in the context of inter-professional working. It is possible that the role protection feelings in our study are suggestive of professional identity threat which is construed when there is a perceived risk of marginalisation or devaluation of the profession’s role or expertise [24]. A key aspect of the PROPEL intervention involved the introduction of inter-professional working, which is defined as ‘a willingness to share and indeed to give up exclusive claims to specialised knowledge and authority if other professional groups can meet patient needs more efficiently and appropriately’ [25], pp 333. Inter-professional working, by definition, implies blurring of professional divisions and clinicians undertaking tasks previously within the domain of other professions [25]. However, blurring of professional roles has been found to threaten professional identity, under certain contextual circumstances, if it is interpreted as an intolerance of professional differences, devaluing of the traditional health professions, or an encroachment on existing roles and scopes of practice [24, 26]. We envisage that these perceptions of devaluation and encroachment acted as what McNeill et al. [24] describe as ‘professional identity faultline’ (a hypothetical dividing line), which, when triggered, increased the salience of professional identities and resulted in heightened perceptions of identity threat, identity conflict and professional divisions. This adds to the examples reported in previous literature of how professional identity threat generated conflict within inter-professional teams, leading to negative outcomes. For instance, resistance has been reported among several professional groups to creation of new roles (e.g. ‘generic healthcare worker’ [27] or ‘independent nurse practitioner’ [28] or ‘nursing role expansion’ [29, 30] and professional conflict has been reported in the context of overlapping roles (e.g. primary healthcare teams [31]; physiotherapy and occupational therapy [32]).

It has been suggested that although professional identity faultlines are always present in inter-professional contexts, they are only triggered in certain circumstances [33]. This means that if strategies can be identified that prevent activation of these faultlines, then inter-professional working can be facilitated and its benefits can be secured [26]. Our study identified the contexts which intensified or moderated the effects of strong professional identity faultlines. We found that professional identity threat was mainly activated and intensified in contexts with adequate specialist capacity and lower need of service expansion. The threat was less salient in contexts characterised by staff shortages, rural and remote geography and service inaccessibility and was in fact counteracted by the heightened perceptions of service need which moderated the feelings of threat and role protection. Our study suggests how emphasising super-ordinate team goals [33] such as increasing the ‘perceptions of service need’ among staff and management may be a useful strategy in future implementation efforts, while carefully managing role-protection feelings and alleviating (mainly specialist physiotherapist) staff’s concerns about compromising care quality through effective leadership and active change management practices [26, 34].

Second, in sites which trained different staff types, the staff who had some level of prior knowledge of women’s health issues and of the physiology of the pelvic region were more comfortable with this new role and were more likely to feel confident following the one day training. Adequate level of training, along with ongoing support from specialist staff, enabled the newly trained staff to provide a high level of tailored, person-centred POP care. This resulted in a positive care experience for women. Staff that were unprepared for the new role and PFMT training and were inadequately supported in practice, were less likely to be confident in the new skills. This led to a less positive care experience for women, despite some objective improvements in symptoms. ‘Staff’s confidence in their capabilities and skills’ and ‘access to mentoring support’ have been highlighted in previous literature as important factors for successful implementation of role expansion, particularly in nursing [30, 35, 36]. When implementing changes involving role expansion, careful selection of staff for expanded roles is important, in addition to training and support, to sustain their motivation, confidence and performance. Additionally, involving specialist staff in close supervision and mentoring of the expanded roles may help protect the specialist role and help alleviate role-protection feelings, potentially through fostering an ‘inter-professional team identity’ [33].

Finally, implementation of the new models was found to be seamless when organisational support (e.g. caseload autonomy, dedicated rooms and time, adequate staffing) was made available to newly trained staff to incorporate PFMT delivery in their role and core work, but when such support was lacking (e.g. inadequate staffing, unchanged workload, lack of dedicated facilities and time), PFMT delivery was seen as an add-on and low priority and felt to cause a degree of discontent among the wider team. This resulted in increased pressure on staff in new roles, overall service disruption and service delays. This finding supports and adds to the literature emphasising the importance of organisational, managerial and team support for the successful development and implementation of new or expanded roles [30, 35, 37].

### Strengths and limitations

Strengths of this study include inclusion of five diverse settings, from those sites which found it easy to implement to those which struggled to establish a different care pathway. The study also benefitted from a large sample of women and a large and relatively consistent sample of healthcare professionals and managers. The study however has some limitations. First, there was a lack of longer-term follow-up to explore if the new service provisions were maintained. Second, there were fewer staff interviews than planned in some areas as these were difficult to obtain (due to staff turnover, unavailability, and logistic issues), despite significant effort. Third, two sites could not be included in the full RE as they were added later to the study to boost recruitment targets. However, these added valuable, confirmatory data to the original RE.

#### Implications for practice and research

The evidence from the PROPEL project supports training a broader range of healthcare professionals with an interest in women’s health and/or with a knowledge of physiology/body muscles to deliver PFMT to women. Delivery of PFMT by other clinicians could be supported by specialist physiotherapists undertaking triage of women to determine their suitability for PFMT. The role of the specialist physiotherapist could then be enhanced, to provide education and support to other healthcare professionals to enable them to safely deliver PFMT to women whilst managing more complex cases of POP themselves. Increasing the use of, and referral for, PFMT as a first-line treatment could be facilitated by improved multi-disciplinary team working across Urogynaecology services and improved communication with primary care. A review of existing pathways to PFMT could identify areas for improvement. Primary care referrals for PFMT as a first-line treatment for POP would likely be increased with more awareness raising (of POP and PFMT) and education for GPs and other primary healthcare professionals.

Prevention of POP would certainly be ideal and opportunities for this exist in primary and maternity care sectors. Further work would be needed to devise appropriate behaviour change strategies for implementation of prevention interventions in these settings. Early detection of POP in primary care also needs to be improved. Existing evidence highlights several barriers to women’s seeking help and receiving timely diagnosis for symptoms of POP, including women-related factors (e.g. lack of knowledge about symptoms, shame and embarrassment, difficulty disclosing symptoms) as well as professional-related factors (such as professional’s lack of knowledge about prolapse, dismissive response to women’s symptoms and lack of proactive intervention) [6, 38, 39]. This suggests that behaviour change interventions that address these barriers among women and primary care professionals will be required to improve early detection of POP. Future research could focus on development, evaluation and roll out of prevention and early detection interventions for POP.

In summary, the findings suggest that successful implementation of PFMT for prolapse requires a) adequate training tailored to differential needs of skill-mix; b) increased awareness of PFMT and behaviour change for early detection among women, GPs and other healthcare practitioners; c) well-coordinated and flexible referral systems; d) wider (multidisciplinary) team support/buy-in for PFMT delivery through different staff skill; e) organisational and managerial support (in terms of resources, training, time, autonomy and staffing) with effective leadership; and f) balancing of likely feelings of role protection with the population needs.

## Conclusion

The RE combined with the robust outcomes data confirms that PFMT can be successfully delivered using a range of staff/skill mixes and in different NHS settings and that outcomes are not compromised by different delivery models. This study supports further roll-out of delivery of PFMT (beyond delivery by specialist physiotherapists) by clinicians (nurses, other physiotherapists) who have an interest in women’s health. This needs to be carefully implemented ensuring adequate levels of training and ongoing support from specialist physiotherapists, multi-disciplinary teams and management.

## Supplementary information

**Additional file 1: Supplementary File 1**: Interview topic guides.

**Additional file 2: Supplementary File 2**: Site-specific Context-Mechanism-Outcome Configurations developed in Phase 2 of the Realist Evaluation.

## Data Availability

The datasets used and/or analysed for this study will be available from the corresponding author on reasonable request and in accordance with consent and ethical approval.

## Abbreviations

AHP: Allied Health Professional
CMO: Context, Mechanism, Outcome
GP: General Practitioner
MSK: Musculoskeletal
PFMT: Pelvic Floor Muscle Training
POGP: Pelvic, Obstetric and Gynaecological Physiotherapy group
POP: Pelvic Organ Prolapse
POPPY: Pelvic Organ Prolapse PhysiotherapY
RE: Realist Evaluation
RE-AIM: Reach, Effectiveness, Adoption, Impact, Maintenance
UK: United Kingdom
NHS: National Health Service
