# Supplementary material for: Implementing pelvic floor muscle training for women with pelvic organ prolapse: a realist evaluation of different delivery models
Source: BMC Health Serv Res. 2020 Oct 1;20:910. doi: 10.1186/s12913-020-05748-8 (PMC7528592; doi:10.1186/s12913-020-05748-8)
Supplement: Supplementary file 1 — Additional file 1: Supplementary File 1: Interview topic guides. [file 12913_2020_5748_MOESM1_ESM.docx]

**Interview Topic Guides**

**Managers and NHS Staff Interview Topic Guides**

**Questions in Round 1 interview**

General introduction to the purpose of the interview. General introduction by the interviewee of their managerial or clinical role in relation to urogynaecological or physiotherapy or primary care services.

- How is the care of POP organised in your area?
- What is/has/or will be your role in developing the new PFMT service?
- What is the need for this change in your area? What has prompted this change in service model?
- What is the general attitude towards plans for a new service involving non-specialist physiotherapists to deliver PFMT? – Reactions by specialist physios, by the selected staff groups, by their managers?
- How easy/ difficult has it been to get people round the table and agree to what needs to be done?
- From your perspective, what are the likely ideas for implementing PFMT in your area? What changes in the service delivery/models are being proposed?
- Why do you think this will be the way forward?
- Wat may be the circumstances in which this new model can be successfully implemented? (e.g. resources, capacity, training, funding, buy-in from different stakeholders etc.)
- What barriers do you anticipate to training new staff groups to deliver PFMT? Any facilitators?
- If the new service model for delivering PFMT is implemented in practice, could you describe me how and why it would help improve outcomes for women with POP?

**Additional questions in Round 2 interview**

- What specific plans have been agreed at this stage? Who will deliver PFMT?
- What are the next steps for operationalising these plans/decisions – who is taking what forward? Who will consult and liaise with staff groups?
- Are there any organisational or managerial problems or issues that have arisen? How have these been managed?
- Do you anticipate any impact on other staff groups or services when the PFMT service is implemented?
- Have other stakeholders (other staff groups, or services incl. GPs) been consulted or informed about the proposed PFMT service? What are their views on the new service?

**Questions in Round 3 interview**

- General introduction to the purpose of the interview.
- What is/has been your role in developing the new PFMT service?
- How is the new service is operating: how are referrals made to the service, how is work allocated/cascaded to trained PFMT staff? How smoothly is the new service operating? How appropriate are the referrals being made? Are those delivering the PFMT comfortable with their new roles and the training they have received? Do they require additional support?
- Has the training enhanced the new staff’s skill set in terms of assessment, judgements about prolapse severity, and PFMT delivery?
- Are there any resource issues for the new service? (Equipment, admin support etc.)
- Are there any organisational or managerial problems or issues that have arisen? How have these been managed? Any issues around resources/funding/facilities?
- Any impact on other staff groups or services when the PFMT service is implemented?
- Has the awareness of PFMT increased among GPs and women since the PFMT service is implemented? Have the GP referrals to PFMT service gone up?
- Any impact the services are having on women or how they have been received?
- Any other problems or issues that have arisen? Any particular aspects that you think are working well or lessons learned?

**Additional questions in Round 4 interview**

- What is your overall impression about your local implementation of PFMT for women? (Successful? If so what have been the key drivers for success)
- Do they anticipate and changes or modifications are needed? If they were to begin again to develop a service would it still look the same?
- Will the services be maintained, changed or expanded?

**Staff Delivering PFMT Interview Topic Guides**

**Questions in Round 2 interview**

- General introduction to the purpose of the interview. General introduction by the interviewee of their usual clinical role in relation to urogynaecology or physiotherapy or women’s health.
- What do you currently know about plans to involve you in delivering PFMT services to women in your area to manage pelvic organ prolapse?
- What is your general opinion/thoughts about these plans?
- What do you think about taking on this new role? Do you have any concerns? How might these be overcome?
- What are your thoughts on the training you will need? Any concerns? What barriers do you anticipate in undertaking this training?
- What barriers do you anticipate to setting up the new service (referral processes, triage if appropriate, location, and admin or resources)?
- What impact will this have on your professional role? (positive or negative?) On your workload? On others’ roles or other services
- Is this new service model going to work for everyone? Could you describe me the types of people and places where this model will be more effective?
- If the new service model for delivering PFMT, which involves your professional group, is implemented in practice, could you describe me how it would help improve outcomes for women with POP?

**Questions in Round 3 interview**

- General introduction to the purpose of the interview.
- In general how do you think things are going with delivering PFMT to women? How do you feel about delivering PFMT?
- Was the training adequate or is more support required? Has this been addressed?
- How has the training made a difference to your clinical skills and practice?
- Any problems in setting up/delivering the new service (referral processes, triage if appropriate, location, and admin or resources)?
- Do you think there has been any change in awareness of GPs/women about PFMT?
- How many women have they seen/treated with PFMT? Were referrals they received appropriate? Where do the referrals come from? How do they see future patterns of referral?
- How has this role (delivering PFMT) impacted on your other work? Are their implications? How supported do you feel by the organisation/management in delivering this service? Any impact on their workload, others’ work and services, and women?

**Additional questions in Round 4 interview**

- In general how do you think things are going with delivering PFMT to women? How do you feel now about delivering PFMT?
- Are there any on-going problems: Training and support issues or problems in delivering the new service (referral processes, triage if appropriate, location, and admin or resources)?
- What do you feel have been the key lessons learned so far? What could have been done differently? What were barriers? What were facilitators? Any recommendations for change and how these might be taken forward?
- Any impact on others’ role, other services and women?
- Do they see delivering PFMT as now an embedded part of their role/job function? Do they see the service continuing?

**Women’s Interview Topic Guides**

**Questions in Round 3 interview**

- General discussion of any previous experience with PFMT exercises and their views of PFMT in general and what their initial reaction was to being offered PFMT as a treatment option. Who was it offered by?
- General discussion of previous experience of attending services or receiving treatment for prolapse and their perceptions of services.
- What are their personal expectations regarding PFMT as a treatment for their prolapse? Any anticipated problems in engaging with PFMT
- What are their perceptions of the arrangements for delivery of PFMT (information received, location/access, number of sessions). What might make it easier to attend PFMT services? (location, format of delivery)
- What are their perceptions of PFMT so far? Have they experienced any problems/ issues? Do they have any comments about how these might be addressed?
- How are they feeling about the remainder of their PFMT treatment?
- Any ideas or further feedback they have for local planning teams in relation to the delivery of PFMT?

**Questions in Round 4 interview**

- General reminder about the first interview and that the aim of this interview is to follow-up on their experiences of PFMT now they reached the end of their treatment period (or what would have been the end for women who may not have completed/dropped out).
- What has been their overall impression of PFMT and its delivery to them? Pick up on and introduce further questioning around the issues that are important to the woman being interviewed.
- Did they attend all appointments: if not why not?
- Did they find they could do the exercises at home/outside of the PFMT appointments? What factors helped or hindered self-directed exercises?
- Do they feel they understood PFMT and whether they had the correct technique?
- Any problems/ issues in using PFMT? Any comments about how these might be addressed by services?
- Any problems/issues with the PFMT service? (staff, location, other delivery aspects).
- What are their perceptions now of the arrangements for delivery of PFMT (information received, location/access, number of sessions). What might make it easier to attend PFMT services? (location, format of delivery).
- Do they feel PFMT has had any impact on their prolapse? If yes -In what ways? If no- do they have any ideas about why that might be?
- Do they think they will continue to use PFMT techniques/exercises? If not why not?
- Any ideas or further feedback they have for local planning teams in relation to the delivery of PFMT?
