# Supplementary material for: Implementing pelvic floor muscle training for women with pelvic organ prolapse: a realist evaluation of different delivery models
Source: BMC Health Serv Res. 2020 Oct 1;20:910. doi: 10.1186/s12913-020-05748-8 (PMC7528592; doi:10.1186/s12913-020-05748-8)
Supplement: Supplementary file 2 — Additional file 2: Supplementary File 2: Site-specific Context-Mechanism-Outcome Configurations developed in Phase 2 of the Realist Evaluation. [file 12913_2020_5748_MOESM2_ESM.docx]

## **Additional File 2**

## **Site-specific Context-Mechanism-Outcome Configurations developed in Phase 2 of the Realist Evaluation**

Table 1: Impact of PROPEL intervention on the reach of PFMT

| **Context** | **Actor** | **Mechanism** | **Outcome** |
| --- | --- | --- | --- |
| **Site A** | | | |
| - High level of training among specialist physios - Adequate number of specialist physios in community and hospital - Team with vision and objectives | Specialist physiotherapists  GPs  Consultants | - No change in referral processes, or delivery of PFMT. - Direct referrals routes from consultants/GPs to specialist physios (and back). - Good co-ordination and communication among referral team (GPs, consultants and Specialist physiotherapists) | - No change in number and profile of patient group treated. |
| - Staff sickness and absence increased during PROPEL implementation | Specialist physiotherapists | - Increased waiting times for PFMT appointments | - Slow turnover of patients |
| **Site B** | | | |
| - Special interest/keen desire among staff for PFMT - Prior training/involvement in assessing/treating incontinence, performing internal examinations - Better understanding of muscle training and exercise prescription, due to physiotherapy training - Good support network | Newly trained MSK physiotherapists  Newly trained continence link nurse (CLN) | PROPEL training enhanced knowledge, clinical skills and confidence.   - Uptake of training was easier and faster among these staff which eased their transition to PFMT delivery. - Staff able to function independently without needing much support from specialists. | - Reach of PFMT widened to the local community, with treatment being available closer to women’s homes. - Saved women long trips, travelling time and inconvenience |
| - No prior gynaecology/pelvic floor experience - Participation in PROPEL was not voluntary – ‘told to get involved’. - High level of existing workload (For ANP) - Lack of management support to develop continence teams/dedicated time - Nurses’ training needs were different from physiotherapists, which were not addressed. They also did not have same level of autonomy as MSK/general physios over their caseload (remain at ‘beck and call’ of GPs). | Advanced nurse practitioner (ANP) and community nurse for continence (CN) | - ANP perceived mismatch between her expectations of the role and what it actually involved. - ANP unable to juggle PFMT caseload due to existing workload - CN overwhelmed by training, felt it was pitched at higher level. Insufficient for those with no prior experience. Felt less competent and lost confidence. - Perception that assessment/treatment of POP is outwith the scope of community nurses. Internal assessments deemed inappropriate in home settings and PFMT deemed inappropriate for people with dementia- both of which form a substantial part of community nurse’s role. | - Early withdrawal from PROPEL - Unable to provide PFMT in local community - Housebound patients lose out on the PFMT service. - Increased implementation burden on specialist physios |
| - Awareness raising/publicity among GPs by specialist physios (prior to and during PROPEL via emails, talks) - Self-referral form redesigned and distributed to GP practices | Specialist physiotherapists  GPs | - GPs reported to be more aware of PFMT service (but not in all areas of the site) - Women reported change in GPs’ actions and attitude to PFMT – GPs were more positive about PFMT, referred actively and quickly than before. This positively influenced women’s expectations. - Women recommending friends to access PFMT through self-referral. | - Perceived increase in GP referrals to new service - Referrals for continence treatment, not just management - Increased local visibility and awareness of PFMT service. Service now more connected and communicated with. |
| - Limited capacity of newly trained staff - Long waiting times for PROPEL appointments | Specialist physios | - Potentially eligible patients not referred to PROPEL to avoid long waiting times (2 months in PROPEL, 3 weeks for specialist physios clinics). | - Referrals to PROPEL restricted in some areas |
| **Site C** | | | |
| - Shortage of doctors & increased patient volume posed workload issues - Busy clinics with many junior doctors, less exposed and experienced in gynaecology | Doctors/consultants | - Referrals to PFMT not a priority, often forgotten. Difficulty talking to patients about PROPEL in busy clinics. - Clinicians not referring to PFMT adequately – remained uninterested and distant to PROPEL. - Referrals not opened to all urogynaecology team (e.g. nurses and physios who see UI or prolapse patients) - Research nurses were brought in to minimise requirements placed on doctors for patient recruitment and referrals. | - Very few referrals to newly trained staff during study period. Limited the reach of PFMT to wider group of women. |
| - Lack of PFMT awareness among GPs and women | GPs  Women | - No efforts were dedicated to raise awareness among GPs, women or communities. - GPs continued to refer to gynaecology. | - Fewer referrals to PROPEL staff. PFMT failed to reach to wider local community. |
| - Less perceived need for PROPEL intervention - Complex care needs of women | Specialist physiotherapists  Higher grade nurses | - Perception of threat to specialist role - Protective feelings about specialist profession - Reluctance to train lower grade nurses or physiotherapists - Concerns that staff of lower banding provided with ad-hoc training may negatively impact on women’s care as their complex care needs may not be addressed appropriately | - Prevention of community model being adopted - Failure to extend reach of PFMT in community - Care not available closer to home. |
| **Site D** **(light touch site)** | | | |
| - Variety of referrals routes from a range of HCPs (GPs; Consultants, Midwives, Health Visitors, MSK physios) - Good relationship with consultants and other HCPs - Good awareness of service - Service promoting benefits of PFMT, holds education sessions about role of physio team to midwives and continence team | Implementation team | - PFMT rolled out to whole MSK physio team - Clinical research team emailed gynaecology to encourage referrals for PROPEL/PFMT - Increased referrals for PFMT from MSK physios | - Additional resources created for women’s health - Widened reach of PFMT through increased staff-skill mix - Increased knowledge of physio team more generally |
| - Service culture favourable to implementation; Staff input to service improvement encouraged - Some non-specialist physios had started PFMT prior to PROPEL; received prior training in prolapse management, internals, urinary incontinence - Perception that physios work in a good supportive environment with informal peer support - Lead physio passionate about women’s health and motivates staff | Newly trained MSK physiotherapists | - PROPEL training appropriate to those with little or no experience but also a refresher for others - Referrals could be made to other staff for more complex cases - Maintained confidence after training - Active support from lead physio and senior physio colleagues for PROPEL | - Increased provision of PFMT - PROPEL seen as the new gold standard for prolapse management and PFMT |
| **Site E (light touch site)** | | | |
| - Prior to PROPEL, limited provision of PFMT due to lack of awareness and training of HCPs - Keen interest in widening access to PFMT - Already providing PFMT for prolapse through the women’s health team involving general physiotherapists. - Excellent communication between referrers and staff providing PFMT - Variety of referral routes and close working with gynaecology, urology, colorectal, GP, continence teams - Consultant pro physiotherapy | MSK physiotherapists  Urogynaecology nurses | - PROPEL training augmented and consolidated staff’s clinical skills - Improved understanding of assessment of prolapse - Training for PROPEL appropriate for level of staff knowledge and skills (including nursing staff) but also very thorough - Adequate on-going support provided by specialist physiotherapist. | - Widened reach of PFMT though widened skill mix - Perceived increase in status of physios proving PFMT within staff team - Greater awareness of staff about PFMT as treatment option |

Table 2: Impact of PROPEL intervention on women’s symptoms, quality of life and care experience

| **Context** | **Actor** | **Mechanism** | **Outcome** |
| --- | --- | --- | --- |
| **Site A** | | | |
| POPPY trial site, high level of training and experience among specialist physiotherapists | Women receiving PFMT  Specialist Physiotherapists | - Physios provide a structured and tailored programme of PFMT treatment (with clear plans), teach correct PFMT technique - Good and clear explanations by physios , lifestyle advice and information - Increased ownership of treatment among women and awareness of their own role in treatment. Women feel accountable to physiotherapists (knowing that their progress will be assessed). Good adherence to PFMT appointments and exercises | - Perceived improvement in symptoms (muscle control, improved incontinence and quality of life) - Improved control over symptoms - Improved mental wellbeing |
| Specialist physios have autonomy over appointment system (can make their own appointments) | Women receiving PFMT  Specialist Physiotherapists | - No feeling of being rushed, accessible location for appointments, flexibility with times - Physios seen as highly skilled and knowledgeable, approachable, relaxed, put women at ease, takes embarrassment away, provide follow-up - Women can and do discuss other treatment options with physios - Personalised approach to treatment planning (setting personal expectations and goals) rather than stereotypical treatment. | - Positive experience of care - High satisfaction with care |
| **Site B** | | | |
| - Long standing and keen interest in continence problems and receiving PFMT training - Anticipation of seeing more prolapse patients due to the CLN role | Newly trained continence link nurse (CLN) | - Training increased the understanding of prolapse and provided better assessment skills - Now better understands the relevance of lifestyle questions asked to patients during assessment - Undertakes holistic assessment of pelvic floor issues (e.g. incontinence). Extended and applied the knowledge and PFMT training to wider set of patients (e.g. incontinence), not just those with prolapse. - Adequate support given by specialist physiotherapists | - Improved practice - Improved detection and outcomes for a wide range of pelvic health issues |
| - Management support in terms of facilities, staff backfill, dedicated time. - Fewer, manageable referrals in general | Newly trained MSK physiotherapists and continence link nurse (CLN) | - Training enabled staff to provide better explanations to women about prolapse and PFMT - The ability to perform internal assessments made patients engage more with treatment. Women benefitted from feedback on muscle strength through internals. - Staff corrected women’s PFMT technique; encouraged them to develop more disciplined personalised PFMT routine - Greater realisation among women of the importance of PFMT to avoid symptom worsening/surgery/pessary - Greater ownership of treatment by women; improved adherence to PFMT - Improved bladder control, regained confidence to resume previous activities, physiotherapists suggested alternative ways of keeping fit. - Women feeling empowered to deal with problem; reduced fear of symptoms worsening - Specialist physiotherapists provided on-going support | - Improved prolapse symptoms (although not resolved completely); feel improved muscle strength, reduced discomfort.  - Improved symptoms of incontinence   - Improved quality of life   increased self-efficacy and control over symptoms  - Experience/self-report of improvement was greater than measurable improvement |
| - Increased confidence and skill through PROPEL training, adequate support from specialist physiotherapists and management. |  | - Physios made women comfortable and feel relaxed, spent more time explaining, very discrete while doing internals. - Physios were perceived to be pleasant and approachable, understanding, thorough, informative, and professional. | - Positive experience of care - Adherence to PFMT appointments |
| **Site C** | | | |
| - Delays in management decisions about place of PFMT delivery - Long gap between PFMT delivery training and actual treatment delivery | Newly trained PROPEL nurses | - Initial loss of confidence in delivering PFMT - Perception that they were ‘thrown in the deep end’, felt apprehensive about seeing first patients - Low confidence sensed by women, staff seen as ‘finding their way’ | - Perceived lack of improvement in symptoms. - However, greater clinically evidenced improvement in symptoms. |
| - Specialist physios and newly trained PROPEL nurses located separately - Specialist physios not actively involved in PROPEL implementation - Specialist clinics busy; part-time working meant less time and staff available to provide support | Newly trained PROPEL nurses  Specialist Physios | - Physios not providing active support/PROPEL nurses not requesting support - Misunderstanding about need for/availability of support (Physios felt nurses competent and didn’t need support; nurses felt physios busy so didn’t ask for support) - Support limited to observing physio clinic and physio’s check on delivery for first patient - Nurses resorted to doing joint clinics to support each other, which boosted their confidence - Nurses perception that no one else to give support |  |
| - Despite women being motivated to avoid surgery - Despite having an open mind to PFMT | Women receiving PFMT within PROPEL | - Difficulty remembering to do exercises (no written info given), difficulty adhering to lifestyle advice - Disillusionment as expectations didn’t match benefit | - No perceived improvement in symptoms - Surgery needed and planned |
| **Site D (light touch site)** | | | |
| - Implementation support for PROPEL from managers and colleagues - Perception that physios work in a good supportive environment with informal peer support - Clinical assessment service for suitability of referral before allocation to PFMT/PROPEL | Specialist physiotherapists,  Managers,  Wider physio teams | - PROPEL training led staff to offer better explanations to patients; better results from internal examinations - Staff were better able to ask appropriate questions and provide education relating to POP/PFMT to women - Staff now applying PFMT to women other issues e.g. lower back pain/incontinence - Helped improve compliance and confidence among women - Active support from senior physio colleagues for PROPEL | - Reported reduction in need for surgery by last PFMT session; women planning to discuss cancellation of surgery with consultant - Perceived improvement in prolapse symptoms , improved QoL - Positive feedback from women following treatment |
| **Site E (light touch site)** | | | |
| - Good peer support available to PROPEL staff - Management support for implementation (availability of time, staff, rooms, facilities etc.) | Newly trained staff;  Women receiving PFMT | - Increased provision of information about PFMT and prolapse - Advice given about lifestyle - e.g. bowel, constipation, exercise - Improved understanding among women about prolapse, PFMT, lifestyle - Greater compliance with PFMT among women - Improved coping among women with their symptoms - Active support from specialist physiotherapists | - Clinical as well as subjective improvement in symptoms, quality of life - Subjective improvement greater than actual improvement on occasions; women felt symptoms had reduced. |

Table 3: Impact of PROPEL intervention on service delivery

| **Context** | **Actor** | **Mechanism** | **Outcome** |
| --- | --- | --- | --- |
| **Site A** | | | |
| - Additional paperwork for PROPEL - Added aspect of recruitment of women to study (although no change in service delivery, women needed to consent for data collection) | Specialist Physiotherapists | - Staff struggling with additional, unfamiliar paperwork - Extra work for staff as they had to make adaptations to recruitment processes to increase recruitment (e.g. changing letter wording, giving study info face-to-face at 1^st^ appointment, telephoning women prior to sending out study info in post). Perceived as time-consuming. | - Increased anxiety and workload |
| **Site B** | | | |
| - Delays in patient recruitment to PROPEL and inflexibility of electronic patient booking systems meant that time slots allocated to PROPEL patients were unfilled. | Newly trained MSK physiotherapists | - More time required (of MSK and specialist physios) to manage patient appointment diary - Perceived as inefficient use of time - Unfilled slots couldn’t be re-allocated to routine MSK patients; time spent in other admin work instead. | - Perceived additional workload relating to patient appointments. - Perception of delays in MSK patients being treated. |
| Existing demands on MSK physio service, busy staff | Other MSK colleagues | - Feeling that MSK colleagues may perceive additional workload for them as PROPEL physios are providing PFMT. | - Doubts about continued tacit support from MSK colleagues |
| - Limited capacity of newly trained staff - Delays in patient recruitment to PROPEL and inflexibility of electronic patient booking systems. | Specialist physiotherapists | - Potentially eligible patients not referred to PROPEL to avoid long waiting times (2 months in PROPEL, 3 weeks for specialist physios clinics). | - Less reduction in specialist workload - Long waiting times for PROPEL appointments |
| - Autonomy to manage own diary - Tacit management support - Adequately staffed service - Adequate spacing of referrals - Fewer patients in general | Newly trained MSK physiotherapists | - Were able to juggle their caseload to fit PROPEL patients. - Were able to do more PROPEL appointments and less MSK appointments within work schedule | - No detrimental impact on workload - PFMT delivery was adopted into practice to some extent |
| **Site C** | | | |
| - PROPEL nurses worked in Urogynaecology surgical ward - The ward was short staffed and busy. Any staff away from the ward had a knock on effect on staffing and patient care - PROPEL nurses had management responsibilities on surgical ward, no backfill available - Little flexibility in ward staff cover and off-duty arrangements | PROPEL nurses  Other surgical ward nurses | - Ward planning became difficult due to ad hoc nature of PROPEL appointments - Upset/unhappy ward colleagues when PROPEL nurses left ward - Struggle for PROPEL nurses to juggle two roles. This was sensed by women too. - Had to sometimes deliver PROPEL on their days off | - Increased pressure on PROPEL nurses - Disturbed ward organisation - PROPEL nurses decided to stop at 8 patients. |
| - Ward context and facilities not conducive to PFMT delivery - Only one private room available, rest were 2-4 bedded bays - Clinic rooms always busy - Post-op ward, focus on recovery. |  | - Difficulty finding clinic spaces - Difficulty delivering PFMT as time and space were constrained. - PFMT was not given priority in this setting - Nurses perception that no one else to give support | - Doubts surrounding appropriateness of the setting and context to PFMT delivery - Doubts surrounding usefulness of training to post-op nurses |
| - High existing workload | PROPEL nurses  Ward clerk/admin | - Ward clerk brought in to support PROPEL patient administration despite own workload pressure. PROPEL tasks very occasionally forgotten/delayed - Nurses set own appointment times with women to ensure clarity - Ward manager/sister stepped in to support, providing cover for PROPEL nurses | - Reduced pressure on PROPEL nurses - Prevented negative impact on patient care/ward |
| **Site D (light touch site)** | | | |
| - Implementation support for PROPEL from managers and colleagues - Provision of time within work schedule for PROPEL; 1 day per week allocated for PROPEL work - Specific rooms set aside for PROPEL patients - Dedicated time for training and supervision related to PROPEL   Community enterprise model; staff input to service encouraged |  | - Capacity/capability study undertaken by research service with physio service prior to PROPEL implementation which confirmed physio service was suitable for and happy about involvement. - Staff able and allowed to manage their own caseloads and manage their own patient bookings - No perception of additional workload due to PROPEL | - Service coping well with PROPEL implementation - No perceived increase in waiting list for physio from PROPEL - No negative impact on service delivery |
| **Site E (light touch site)** | | | |
| Good implementation support from managers:   - Specific rooms allocated for PROPEL patients - Appointment duration: 1^st^ appointment – 45 mins, subsequent- 30 mins - Provision of time for PROPEL, but patients that physios see anyway - Normal treatment pathway and waiting and follow up times |  | - PFMT seen as core work, not something additional/extra - Shorter waiting times offered for PFMT as a lure for inclusion in PROPEL | - No significant disruption to routine service - No increase in workload |
